# Single-Channel Properties of the ROMK-Pore-Forming Subunit of the Mitochondrial ATP-Sensitive Potassium Channel

**DOI:** 10.3390/ijms20215323

**Published:** 2019-10-25

**Authors:** Michał Laskowski, Bartłomiej Augustynek, Piotr Bednarczyk, Monika Żochowska, Justyna Kalisz, Brian O’Rourke, Adam Szewczyk, Bogusz Kulawiak

**Affiliations:** 1 Laboratory of Intracellular Ion Channels, Nencki Institute of Experimental Biology, Polish Academy of Sciences, 02-093 Warsaw, Poland; michail.laskowski@unimi.it (M.L.); bartlomiej.augustynek@ibmm.unibe.ch (B.A.); m.zochowska@nencki.gov.pl (M.Ż.); j.jedraszko@nencki.gov.pl (J.K.); a.szewczyk@nencki.gov.pl (A.S.); 2 Department of Physics and Biophysics, Institute of Biology, Warsaw University of Life Sciences-SGGW, 02-787 Warsaw, Poland; piotr_bednarczyk@sggw.pl; 3 Division of Cardiology, The Johns Hopkins University School of Medicine, 720 Rutland Avenue, Baltimore, MD, USA; bor@jhmi.edu

**Keywords:** mitochondria, patch-clamp, cardiac muscle, mitochondrial ATP-sensitive potassium channel, renal outer medullary potassium channel, mitochondrial large conductance calcium regulated potassium channel

## Abstract

An increased flux of potassium ions into the mitochondrial matrix through the ATP-sensitive potassium channel (mitoK_ATP_) has been shown to provide protection against ischemia-reperfusion injury. Recently, it was proposed that the mitochondrial-targeted isoform of the renal outer medullary potassium channel (ROMK) protein creates a pore-forming subunit of mitoK_ATP_ in heart mitochondria. Our research focuses on the properties of mitoK_ATP_ from heart-derived H9c2 cells. For the first time, we detected single-channel activity and describe the pharmacology of mitoK_ATP_ in the H9c2 heart-derived cells. The patch-clamping of mitoplasts from wild type (WT) and cells overexpressing ROMK2 revealed the existence of a potassium channel that exhibits the same basic properties previously attributed to mitoK_ATP_. ROMK2 overexpression resulted in a significant increase of mitoK_ATP_ activity. The conductance of both channels in symmetric 150/150 mM KCl was around 97 ± 2 pS in WT cells and 94 ± 3 pS in cells overexpressing ROMK2. The channels were inhibited by 5-hydroxydecanoic acid (a mitoK_ATP_ inhibitor) and by Tertiapin Q (an inhibitor of both the ROMK-type channels and mitoK_ATP_). Additionally, mitoK_ATP_ from cells overexpressing ROMK2 were inhibited by ATP/Mg^2+^ and activated by diazoxide. We used an assay based on proteinase K to examine the topology of the channel in the inner mitochondrial membrane and found that both termini of the protein localized to the mitochondrial matrix. We conclude that the observed activity of the channel formed by the ROMK protein corresponds to the electrophysiological and pharmacological properties of mitoK_ATP_.

## 1. Introduction

Ischemic heart disease remains a major worldwide health concern. The identification of ischemic preconditioning (IPC) as a cardioprotective mechanism has raised the possibility that stimulating the endogenous pathways may protect the heart from ischemic injury [1]. It has been suggested that the maintenance of mitochondrial integrity is crucial in the preconditioning of heart [2] and brain tissue [3]. A large body of evidence has implicated mitochondrial ion channels as significant players in these phenomena [4,5,6]; however the precise role of these channels is still under active investigation [7].

The ATP-sensitive potassium channel (mitoK_ATP_) opening protects cardiac and neuronal tissue against ischemia-reperfusion injury [8,9]. Considerable progress has been made in understanding the signaling mechanisms of cardioprotection [10,11], but understanding the role of mitoK_ATP_ in cardioprotection is still incomplete. In 1991, mitoK_ATP_ was first identified using an electrophysiological approach in rat liver mitochondria [12]. mitoK_ATP_ was later found in other mammalian tissues including the heart [13,14,15], brain [16,17], skeletal muscle [18], skin fibroblasts [19] and renal tissue [20]. The presence of the channel has also been reported in non-mammalians including *Crithidia fasciculate* euglenozoa [21], *Caenorhabditis elegans* nematode [22], *Acanthamoeba castellanii* amoebae [23], and in plants, including *Triticum turgidum* wheat and *Pisum sativum* peas [24].

Until recently, progress in identifying its molecular composition has been limited, despite efforts by many laboratories [7]. MitoK_ATP_ has been determined to be functionally and molecularly distinct from sarcolemmal K_ATP_ channels (sarcK_ATP_), based on the finding that diazoxide was 1800 times more potent in opening mitoK_ATP_ than sarcK_ATP_ [15]. This finding was confirmed by Sato et al. [5], who also described the distinguishing effects of HMR1098 and P1075 on the two channels. Initial studies suggested that the inward rectifying K^+^ channel subunits Kir6.1 or Kir6.2 are pore-forming units of mitoK_ATP_, but later studies did not confirm this hypothesis [25,26,27]. Recently, a proteomic screen, together with pharmacological and genetic manipulation, led Foster et al. [28] to conclude that a splice variant of the renal outer medullary potassium channel (ROMK) is a pore-forming unit of cardiac mitoK_ATP_. In this study, we used mitoplasts from rat heart-derived H9c2 cell lines (WT and overexpressing human ROMK2 isoform of the channel) to study the electrophysiological and pharmacological properties of the mitoK_ATP_. Additionally, we performed experiments in order to clarify the structural topology of the channel formed by the ROMK2 in the inner mitochondrial membrane.

## 2. Results

### 2.1. Ion Channels Observed in H9c2 Mitochondria

**Figure 1:**
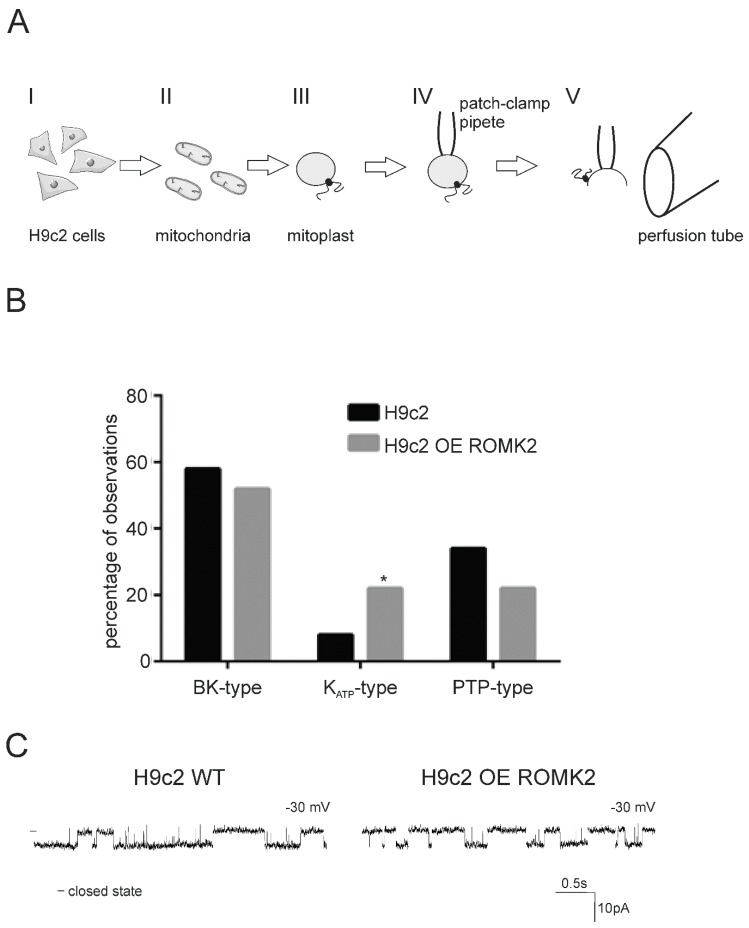
Procedure of the mitochondrial patch-clamp experiment and summary of recorded channel activities. (**A**) Mitoplasts (mitochondria without the outer membrane) were prepared from mitochondria of the H9c2 WT and renal outer medullary potassium channel (ROMK2) overexpressing H9c2 cells (I). Mitochondria were added to the hypotonic solution to induce swelling and outer membrane breakage. Isotonicity was restored by adding a hypertonic solution (II and III). A free-floating mitoplast was attached to a glass pipette (IV). Ion current was measured in an inside–out configuration. The modulators of the channels were added through a perfusion system (V). See more information in the Experimental Procedures section. (**B**) A comparison of mitochondrial ion channel activities from H9c2 WT (black bars) and H9c2 OE ROMK2 (grey bars) cell lines. The analysis was based on *n* = 50 recorded channels (all types) for each cell line. Data were obtained from around 30 independent mitochondrial isolations for each cell line. The number of observed K_ATP_-type channels in mitochondria H9c2 OE ROMK2 was significantly higher. The chi-squared statistic was 3.8431, * *p* < 0.05. (**C**) A comparison of ion channel kinetics classified in Panel B as mitoK_ATP_ in the H9c2 WT cells and H9c2 cells overexpressing ROMK2. Recordings were performed at −30 mV.

The main aim of our study was to describe the mitoK_ATP_ channel present in heart mitochondria and verify our hypothesis suggesting the ROMK protein as a pore forming subunit of this channel. Therefore, we performed a series of electrophysiological experiments with use of isolated mitoplasts (Figure 1A). In our study, we used mitoplasts isolated from both the wild type H9c2 and H9c2 cells overexpressing human ROMK2 (H9c2 OE ROMK2) [28] (for a detailed description of the cell lines, see the Materials and Methods section). The procedure of mitoplast selection was verified by the preparation of a PCR reaction with use of primers which could recognize mitochondrial and nuclear DNA (Figure S1, Tables S1 and S2). We found that all tested vesicles contained mitochondrial but not nuclear DNA.

The channel activity from inner mitochondrial membrane of the cell lines was measured in patch-clamp experiments described in the Materials and Methods section. Performed experiments revealed the existence of few ion channel types in the mitochondria of both cell lines. Based on conductance and kinetics, we classified the observed channels into three groups: (a) large-conductance, calcium-regulated potassium (BK_Ca_-type) channel with a conductance between 250 and 300 pS and brief openings at negative voltage; (b) permeability transition pore (PTP)-type channel with a conductance between 500 and 1000 pS and openings with many sub-states (probably mitochondrial permeability transition pore) (Figure S2); (c) K_ATP_-type with conductance of around 90–100 pS and typical middle openings and closings of the channel (Figure 1B). Based on this classification we found that the number of observed K_ATP_-type channels in mitochondria from H9c2 OE ROMK2 was significantly higher (*p* < 0.05) than in the WT cells. The chi-squared statistic was 3.8431 (based on 50 recordings of all channel types for each cell line).

A direct comparison of mitoK_ATP_ recordings from the H9c2 WT and H9c2 OE ROMK2 cells revealed the existence of channels with very similar kinetics in the mitochondria of both cell lines (Figure 1C).

### 2.2. Identification of the mitoK_ATP_ Channel in Mitoplasts of ROMK Overexpressing H9c2

**Figure 2:**
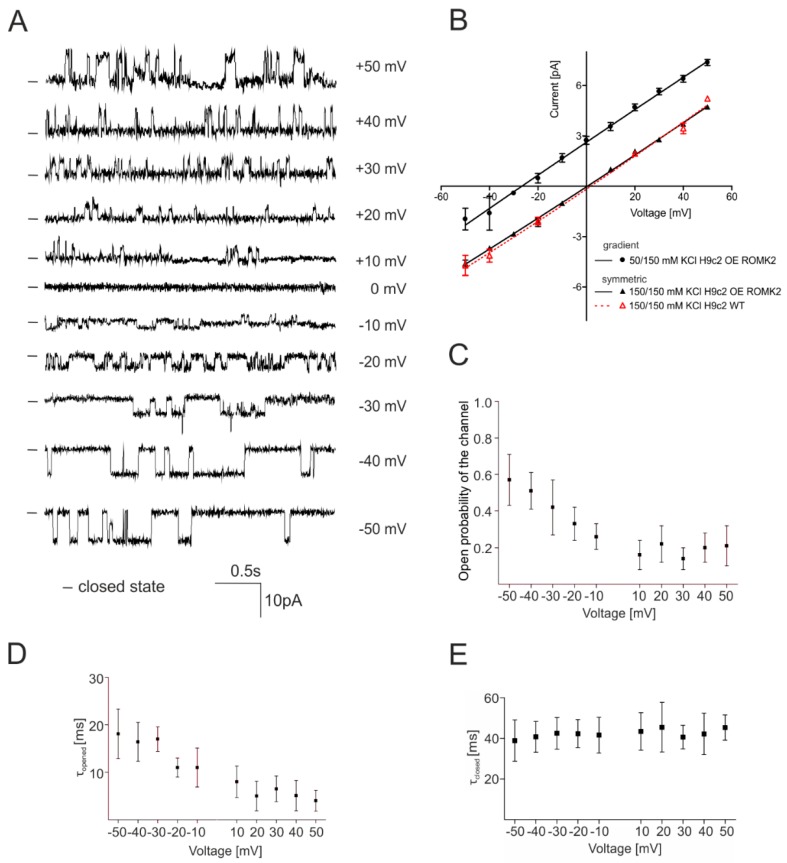
The biophysical properties of mitoK_ATP_ from the inner mitochondrial membrane of the H9c2 OE ROMK2 and H9c2 WT cells using the patch-clamp technique. (**A**) Single-channel recordings in a symmetrical 150/150 mM KCl solution at different voltages of the mitoK_ATP_ channel from the mitochondria of the H9c2 OE ROMK2 cells. (**B**) Current–voltage characteristics of single-channel events in symmetrical 150/150 (solid lines, filled triangles) and asymmetrical 50/150 mM KCl (solid lines, filled circles) of mitoK_ATP_ from the H9c2 OE ROMK2 cells and in symmetrical 150/150 mM KCl of mitoK_ATP_ from the H9c2 WT cells (dashed line, hollowed triangles). (**C**) Channel open probability (P_o_) at different potentials of the channels from the H9c2 OE ROMK2 cells. (**D**) Voltage dependence of the mean channel open time in the symmetrical 150/150 mM KCl solution. (**E**) Voltage dependence of the closed dwell time of the channel from the H9c2 OE ROMK2 cells in a symmetrical 150/150 mM KCl solution. The biophysical properties of the channels were calculated based on individual mitochondrial isolations from independent cell cultures (*n* = 8 in the case of the H9c2 OE ROMK2 cells). Each mitochondrial isolation was performed from 2–3 mln cells. The results are presented as the means ± SD.

A very low frequency of mitoK_ATP_ observations in the H9c2 WT cells stopped us from performing full characteristics of the channel. Initially, we analyzed the current–voltage dependence of both channels. Figure 2A shows the single-channel recordings at different voltages of the channel from the H9c2 OE ROMK2 cells. Based on the current–voltage relationship, the calculated conductance of the channel from the H9c2 OE ROMK2 cells was equal to 94 ± 3 pS in symmetric 150/150 mM KCl (Figure 2B). The conductance of the channel present in mitochondria of the H9c2 WT cells was equal to 97 ± 2 in the same experimental condition. In both cases, rectification was not observed. What is important is that both channels showed an almost identical voltage–current correlation, directly suggesting the same properties and origin of both activities.

Figure 2C–E show the biophysical parameters for the channel activity from the H9c2 OE ROMK2 cells. The open probability (P_o_) increased from 0.21 ± 0.11 at 50 mV to 0.57 ± 0.14 at −50 mV. We observed a voltage dependence on the mean channel opening time (τ_open_), which decreased from 19.2 ± 6.3 ms at −50 mV to 5.1 ± 1.3 ms at 50 mV. The mean mitoK_ATP_ channel closed time (τ_closed_) increased from 38.9 ± 10.2 ms at –50 mV to 45.3 ± 6.2 ms at 50 mV.

### 2.3. Specificity of the mitoK_ATP_ Channel Using the Patch-Clamp Technique

To characterize the specificity of the channel, the ionic current was measured in an asymmetric 50/150 mM KCl solution. In these experiments, the activity of the mitoK_ATP_ channel from the H9c2 OE ROMK2 cells was measured. Figure 2B shows the current-voltage relationship in the asymmetric 50/150 mM KCl solution. The permeability ratios for ions were calculated according to the Goldman–Hodgkin–Katz voltage equation:

$$E_{rev}=\frac{\mathrm{RT}}{\mathrm{zF}}\ln\left(\frac{p_{K}{\left[K\right]}_{o}+p_{Cl}{\left[\mathrm{Cl}\right]}_{in}}{p_{K}{\left[K\right]}_{in}+p_{Cl}{\left[\mathrm{Cl}\right]}_{o}}\right)$$

where E*_rev_* is the reverse potential, R is the gas constant, T is the temperature in Kelvin, z is equal to 1, F is the Faraday constant, p is the permeability of a given ion, and [Cl] and [K] are the respective concentrations of the ion outside (o) (i.e., in the pipette) and inside (in) (i.e., in the chamber). All measurements were carried out at room temperature (25 °C).

This result showed that the observed channel was highly specific for cations. The calculated permeability ratio for K^+^ and Cl^−^ was equal to 46.4 according to the Goldman–Hodgkin–Katz voltage equation. The calculated reverse potential of the channel was –26.8 ± 2.4 mV. The asymmetric solution did not significantly affect channel parameters such as conductance (Figure 2B).

### 2.4. Pharmacological Properties of mitoK_ATP_ Using the Patch-Clamp Technique

**Figure 3:**
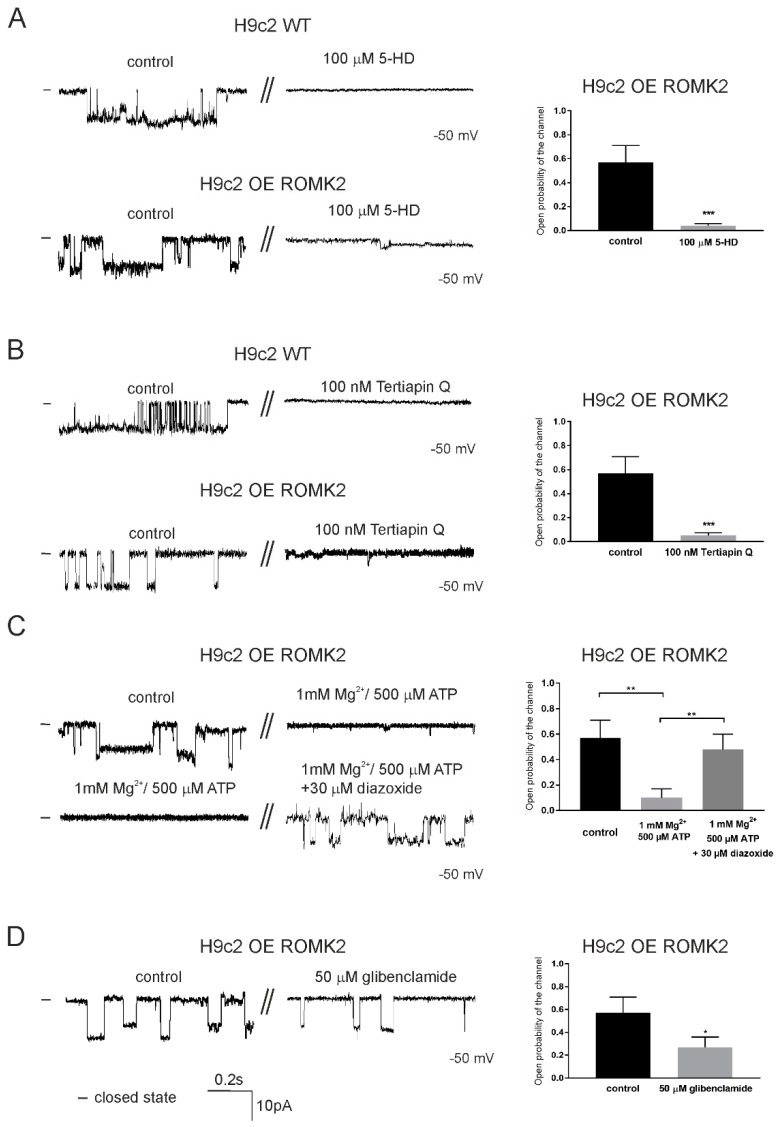
The effects of modulators on the mitoK_ATP_ channel from the inner mitochondrial membrane of the H9c2 OE ROMK2 and WT H9c2 cells. (**A**) Representative traces of the channel from the H9c2 WT cells and H9c2 OE ROMK2 cells at –50 mV under control conditions and after the addition of 100 μM of 5-HD (left panel). On the right panel, an analysis of the P_o_ of the detected channel from the H9c2 OE ROMK2 cells is shown. (**B**) Single-channel recordings of the channel from the H9c2 WT and H9c2 OE ROMK2 cells at –50 mV under control conditions and after the application of 100 nM of Tertiapin Q (left panel). On the right panel, an analysis of the P_o_ of the detected channel from the H9c2 OE ROMK2 cells is shown. (**C**) Representative channel recordings of the channel from the H9c2 OE ROMK2 cells at –50 mV under control conditions and after the addition of 500 μM of ATP and 1 mM of Mg^2+^ (left panel, upper trace). The inhibitory effect was reversed by the addition of 30 μM of diazoxide at –50 mV (left panel, bottom trace). On the right panel, an analysis of the P_o_ of the detected channel under these condition is shown. (**D**) Single channel recordings of the channel from the H9c2 OE ROMK2 cells at –50 mV under control conditions and after the application of 50 μM of glibenclamide (left panel). On the right panel, an analysis of the P_o_ of the detected channel under these condition is shown. The statistical analysis was based on three independent recordings from three independent isolations (*n* = 3). Each mitochondrial isolation was performed from 2–3 mln cells (independent cultures). The results are presented as the means ± SD.* *p* < 0.05, ** *p* < 0.01, or *** *p* < 0.001.

Next, we tested the typical modulators of the mitoK_ATP_ and ROMK channels. Figure 3 illustrates the activity of the channels detected in the inner mitochondrial membrane of the H9c2 OE ROMK2 and H9c2 WT cells under the control conditions and after the application of modulators. All modulators were applied to the matrix site. First, we tested 5-hydroxydecanoic acid (5-HD), a known mitoK_ATP_ inhibitor (Figure 3A).

In both cases, the application of 100 µM of 5-HD resulted in the channel inhibition, as is shown in the original recordings (Figure 3A, left panel). In case of the channel from the H9c2 OE ROMK2 cells, an open probability was calculated from three independent experiments. The application of the inhibitor resulted in a decrease of P_o_ from 0.57 ± 0.14 to 0.040 ± 0.014 at –50 mV (*n* = 3). Similarly, the application of Tertiapin Q (TPNQ), which blocks ROMK channels, also resulted in the inhibition of both channels (Figure 3B, left panel). Similarly to that of 5-HD, the application of TPNQ resulted in a statistically significant decrease of P_o_ (to 0.05 ± 0.02 after inhibitor addition, *n* = 3). Apart from the above, we measured the activity of mitoK_ATP_ from the H9c2 cells overexpressing ROMK2 after the application of 1 mM of Mg^2+^ and 500 µM of ATP (Figure 3C). We found that ATP/Mg^2+^ inhibited the channel activity, and P_o_ decreased to 0.1 ± 0.07 at –50 mV. The addition of 30 µM of diazoxide recovered channel activity, and P_o_ increased to 0.48 ± 0.12 at –50 mV (*n* = 3). The channel was also partially inhibited by 50 µM of glibenclamide (Figure 3D). The channel P_o_ decreased from 0.57 ± 0.14 to 0.27 ± 0.09 at –50 mV (*n* = 3). 

Taken together, the measured single-channel conductance, voltage–dependent regulation, and sensitivity to selective agonists and antagonists strongly indicate that our recordings corresponded to a mitochondrial ATP-sensitive channel as well as a renal outer medullary potassium channel. Based on available data, we conclude that channels from both cells lines classified as mitoK_ATP_ showed very similar biophysical and pharmacological properties. Moreover, we were able to observe mitoK_ATP_ more frequently in the cells overexpressing the ROMK2 protein. These results strongly support the hypothesis that the ROMK protein is the pore-forming subunit of mitoK_ATP_.

### 2.5. Properties of the mitoBK_Ca_ Channel from H9c2 OE ROMK2 Cells

**Figure 4:**
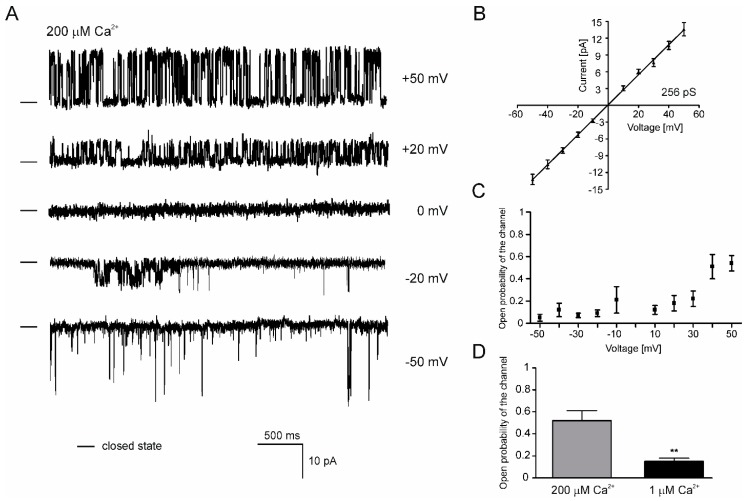
Single channel recordings of the mitoBK_Ca_ (Mitochondrial large-conductance, calcium-regulated potassium) channel of the inner mitochondrial membrane of the H9c2 OE ROMK2 cells. (**A**) Representative single-channel recordings in a symmetrical 150/150 mM KCl solution at different voltages of the mitoBK_Ca_ channel from the ROMK2 overexpressing H9c2 cells. (**B**) Current–voltage characteristics of single-channel events in symmetrical 150/150. The channel conductance was equal to 256 pS. (**C**) Channel open probability at different potentials. (**D**) Data analysis of the channel open probability in the presence of 200 μM (grey bar) and 1 μM (black bar) concentrations of Ca^2+^. The statistical analysis was based on three independent recordings from three independent isolations (*n* = 3). Each mitochondrial isolation was performed from 2–3 mln cells (independent cultures). The results are presented as the means ± SD. ** *p* < 0.01.

Apart from the mitoK_ATP_ in both cell lines, we were able to detect large-conductance Ca^2+^-regulated channel with properties corresponding to well-known mitoBK_Ca_ channels (Figure 1B and Figure 4). The single-channel recordings at different voltages revealed the voltage sensitivity of the channel from the H9c2 OE ROMK2 cells (Figure 4A,C). The activity of the channel manifested by P_o_ was significantly reduced in the negative voltages. Based on the mean of the current-voltage relationship, the calculated conductance of the channel was equal to 256 pS in symmetric 150/150 mM KCl (Figure 4B). Additionally, the channel showed a clear sensitivity to Ca^2+^ ions (Figure 4D). The channel open probability was significantly lower after perfusion with a medium containing a low concentration of Ca^2+^. Based on the presented data, we conclude that properties of this channel correspond to previously described mitoBK_Ca_ channels. Our electrophysiological data are in line with a biochemical analysis that showed the presence of the BK_Ca_ channel subunits in mitochondria of the H9c2 cells [29].

### 2.6. Localization of the ROMK protein after Transient Expression in H9c2 Cells

**Figure 5:**
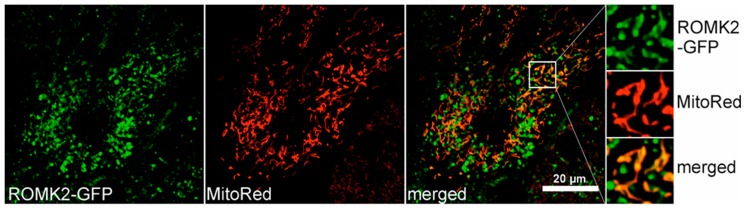
The localization of the ROMK2-GFP protein in H9c2 after transient transfection. Confocal images of the cultured H9c2 WT cells stained with MitoRed to detect mitochondria (red channel) and ROMK2-GFP (green channel). The superimposition of the two signals revealed the mitochondrial localization of the channel in H9c2 WT (orange). Part of the green signal was found to localize to other cellular compartments.

The above electrophysiological data suggest presence of the same mitoK_ATP_ in the mitochondria of both the WT and ROMK2 OE H9c2 cells. To verify our electrophysiological data, we checked the localization of the ROMK2 after the transient transfection of the wild type H9c2 cells. Indeed, the transient transfection of ROMK2 fused with GFP resulted in a clear GFP signal overlap with mitochondrial staining (Figure 5). Furthermore, a part of the ROMK2-GFP fraction was located outside the mitochondria in other cellular fractions most likely as a result of overexpression. The above data confirm the targeting of the ROMK protein to the mitochondria of H9c2 WT and support functional studies previously performed in these cells [28].

### 2.7. Localization in the Inner Mitochondrial Membrane and Possible Mitochondrial Topology of ROMK Protein in H9c2 OE ROMK2 Cells

**Figure 6:**
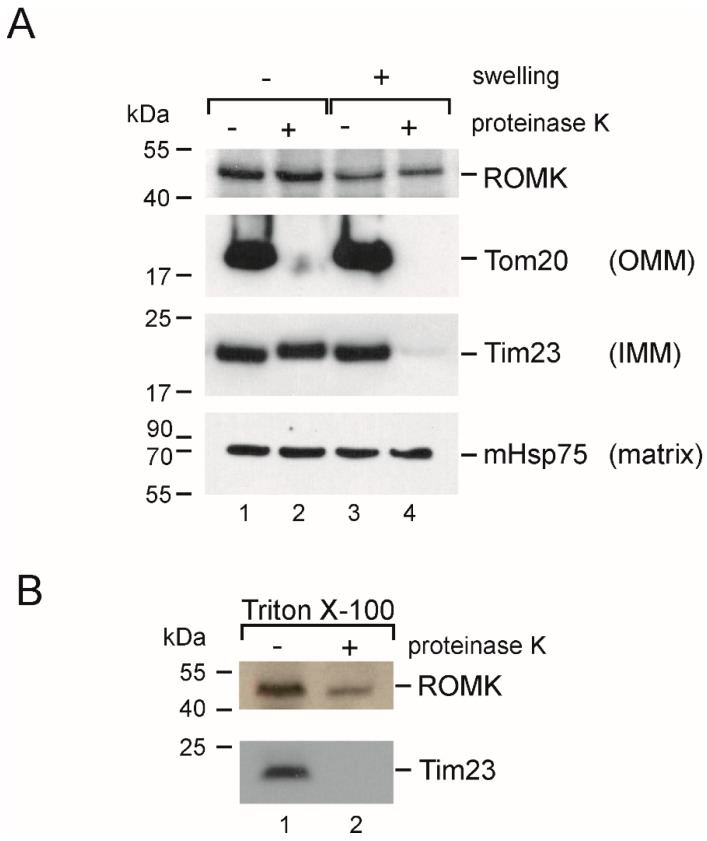
Topology of the mitoK_ATP_ channel from the inner mitochondrial membrane of H9c2 OE ROMK2. (**A**) Western blot analysis after Proteinase K (PK) treatment (OMM—outer mitochondrial membrane; IMM—inner mitochondrial membrane). (**B**) Western blot analysis after the PK treatment of mitochondria solubilized with Triton X-100 (TrX). Each experiment was performed from independent mitochondrial isolation (*n* = 5). Each mitochondrial isolation was performed from 25–30 mln cells.

Finally, we decided to confirm the presence of the ROMK protein in the inner mitochondrial membrane using a Proteinase K (PK) treatment assay (Figure 6). In these experiments, the mitochondrial fraction from the H9c2 OE ROMK2 cells was used. A western blot analysis showed that ROMK was protected against PK digestion in the mitochondrial samples incubated in an isotonic solution (“–swelling”, “+PK”). In these conditions, the mitochondrial outer membrane protects the proteins of the intermembrane space and inner mitochondrial membrane against proteinase K. The parallel staining of the translocase of outer membrane (TOM) complex receptor (Tom20), which is localized in the outer mitochondrial membrane, showed a complete degradation of the protein. In the same fraction, both Tim23 (a control protein with known localization in the mitochondrial inner membrane) and Hsp75 (matrix) were also protected. The mitochondrial swelling and subsequent PK treatment (“+swelling”, “+PK”) resulted in the degradation of both Tom20 and Tim23. On the other hand, both ROMK and Hsp75 remained untreated after the addition of proteinase. This result suggested the limited access of PK to the epitope of ROMK localized in the C-terminal part of the protein even after mitochondrial outer membrane disruption. However, we could observe the partial degradation of ROMK after the solubilization of mitochondria with 0.1% TritonX-100 and subsequent PK treatment (Figure 6B). This result suggests that the observed signal came from a protein localized to the inner mitochondrial membrane. Moreover, since ROMK2 contains two transmembrane segments, our data might suggest that both the N- and C-termini of the protein might be localized in the mitochondrial matrix.

## 3. Discussion

In our study, we used mitoplast patch-clamping to detect mitoK_ATP_ in H9C2 cells. Previous observations concerning mitoK_ATP_ were based on measurements of K^+^ ion flux across the inner mitochondrial membrane or across the liposomal membrane following the partial purification and reconstitution of mitoK_ATP_. These studies clearly revealed that the mitoK_ATP_ opening leads to mitochondrial depolarization, an increase of oxygen consumption and mitochondrial swelling [8]. The single channel properties of the mitoK_ATP_ of liver, lymphocytes, heart or brain mitochondria have been successfully studied by reconstituting the mitochondrial inner membrane into a planar lipid bilayer [13,14,30,31,32] or with a patch-clamp technique [12,19,33]. Various proteins, including Kir6.1 and Kir6.2, have been proposed to form the channel [26,27]; however, a recent study provided strong evidence that the mitoK_ATP_ in cardiac cells is formed by the ROMK protein [28]. This was supported by a report showing that ROMK-forming mitoK_ATP_ also exists in skin fibroblasts [19]. In the present work, the measurements of ion flows across the inner membrane of mitochondria isolated from the cardiac H9c2 cells revealed the presence of mitoK_ATP_ formed by the ROMK protein.

Reported channels have a conductance less than 100 pS, which is close to previous observations [19,30,33,34] but much higher than the values in the 10–30 pS range observed by Mironova and coworkers [14,32]. However, mitoK_ATP_ conductance has often been found to vary. For example, a study describing the electrophysiological properties of the reconstituted human cardiac mitoK_ATP_ reported maximum conductances from ~20 to ~60 pS and >80 pS [35]. The observed conductance of mitoK_ATP_ in human lymphocytes did not exceed 82 pS [33].

ROMK channels expressed in plasma membrane exhibited a conductance of 30–35 pS [36]. However, the ROMK protein is also part of the thick ascending limb potassium secretory channel, which exhibits ROMK-like characteristics and has a conductance of 70 pS [37]. Differences of conductance between plasma membrane and mitochondrial channels can be result of various factors, including the presence of protein partners, posttranslational modifications of the pore forming unit, differences in the lipid composition of mitochondrial inner membrane, and experimental protocol. A similar difference of conductance has also been observed in the case of the BK_Ca_ and mitoBK_Ca_ channels; moreover, the conductance of mitoBK_Ca_ from the same tissue can vary [38].

The mitoK_ATP_ channels in this study did not show rectification under all conditions, which is similar to what was found with the channel from skin fibroblasts [19]. MitoK_ATP_ from bovine heart reconstituted into black lipid membrane (BLM) showed weak rectification but only in a non-symmetrical solution (50/150 mM of KCl) [31]. By contrast, mitoK_ATP_ from lymphocytes recorded with patch-clamp showed rectification in negative voltages [33]. The other biophysical properties of the observed channels match data described in the literature: the decreased opening probability when positive voltage is applied [19,31] and open/close dwell time [35].

The identification of the observed channel as mitoK_ATP_ was also based on its pharmacological properties. The channel recorded in both cell lines was inhibited by 5-HD, which is a key mitoK_ATP_ inhibitor [8]. This assertion is based on the functional measurements of the cardiac channel in cardiomyocytes [39], in a perfused heart [9] and isolated mitochondria [40].

Tertiapin-Q is a known inhibitor of ROMK-type channels [41,42,43] and was shown to inhibit mitoK_ATP_ by Foster et al. [28]. TPNQ was also shown to inhibit the BK_Ca_-type of channels [43,44]; however, the mitoBK_Ca_ channels have significantly different biophysical and pharmacological properties than mitoK_ATP_, as was also reported in this study. What is important is that the mitoBK_Ca_ channel has previously been found in cardiac mitochondria. It has also been shown that activation of the channel protects the heart muscle against injury induced by ischemia/reperfusion [45,46,47]. The presence of both channels in one cell type raises questions about the interplay of both channels in the cytoprotection mechanism and should be studied in future.

A third channel inhibited by TPNQ is the G protein-coupled, inwardly-rectifying potassium channel (GIRK-type, Kir3.x) [43,48]. However, Mg^2+^/ATP did not inhibit the GIRK channels, and ATP increased their activity [49,50]. Because the ROMKs belong to the ATP-inhibited channels [51], we conclude that TPNQ inhibited the ROMK channel.

Additionally, we show that the overexpression of ROMK2 in the H9c2 cells resulted in increase of the mitoK_ATP_ channel recordings with the same kinetics and basic biophysical properties as the H9c2 WT cells. Moreover, the transient transfection of the H9c2 cells with ROMK2-GFP revealed that the channel localized to mitochondria. This observation agrees with previous study showing the localization of the ROMK protein to the cristae in mitochondria from both the heart and the liver [52]. Silencing the ROMK protein resulted in a decrease of ATP-, 5-HD-, and TPNQ-sensitive mitochondrial swelling and thallium uptake in the H9c2 cells [28]. All these findings supports the ROMK protein as a potential component of mitoK_ATP_ in cardiac cells. However, it has been suggested that besides ROMK, there may be another protein that can form this type of channel in mitochondria [53]. This idea is supported by different sensitivity to glibenclamide in both cases. The channel described here was fully inhibited by 5-HD and Mg^2+^/ATP, but it was only partially inhibited by glibenclamide. 

Nevertheless, the partial but statistically significant inhibition of observed mitoK_ATP_ activity by the antidiabetic sulfonylurea glibenclamide may indeed suggest the presence of a mitochondrial inner membrane sulfonylurea receptor (mitoSUR) in addition to the ROMK2 protein. Previous equilibrium binding studies have revealed a single class of low affinity binding sites for sulfonylurea in the inner mitochondrial membrane from beef hearts [54]. Bajgar et al. [16] identified a 63 kD protein labeled with high affinity by BPDIPY-FL-gliburide and proposed that this may be mitoSUR. Several studies have demonstrated that ROMK2 forms a glibenclamide-sensitive K^+^ channel when co-expressed with SUR2B. Moreover, when ROMK2 is expressed alone in *Xenopus laevis* oocytes, it is not sensitive to glibenclamide [55]. Sulfonylurea receptor (SUR2A) subunits have been demonstrated in ventricular myocyte mitochondria [56], but other studies have not supported a role for SUR2B as a component of ROMK2 and have indicated that there is no requirement for an additional subunit to confer glibenclamide sensitivity to ROMK2 [57]. Other studies have suggested that the cystic fibrosis transmembrane conductance regulator (CFTR) may confer ROMK sensitivity to glibenclamide; however, the presence of CFTR in the inner mitochondrial membrane remains unclear [58]. The presence of mitoSUR in the H9c2 cell line has not been clearly demonstrated, and further studies are required to determine the protein partners of ROMK2.

The topology of mitoK_ATP_ in the inner membrane of mitochondria is an important issue. Since the ROMK protein contains two transmembrane domains, the C- and N-termini must be located on the same side of the inner mitochondrial membrane. Inoue et al., [12] concluded that Mg^2+^/ATP inhibits from the matrix. Yarov-Yarovoy et al. [59] concluded that Mg^2+^/ATP inhibits from the cytosolic side of the inner membrane. The measurements of K^+^ fluxes across the inner mitochondrial membrane supported this idea and suggested that the ATP binding domain faces the cytosol (intermembrane space), because the channel inhibited by palmitoyl coenzyme A can be restored by GTP added to an external medium [59]. However, a GDP/GTP transport system in the inner mitochondria membrane has been identified [60,61]. Our mitoplast patch-clamp recordings were performed in an inside–out configuration. This implies that during the channel recording, Mg^2+^/ATP had access to the matrix side of the channel. On the other hand, our recordings revealed the inhibition of the channel by Tertiapin Q. It is known that TPNQ binds to the vestibule of the plasma membrane ROMK channel, which faces the extracellular environment and, thus, the opposite site of the membrane to the nucleotide binding domain [41,42]. Our single channel recording with the application of Tertiapin Q could suggest that this peptide also inhibits the channel from the matrix site. Alternatively, it is possible that the toxin could be partially permeable to the mitochondrial membrane, which would allow it to block the pore in a more conventional way. 

A similar effect of the unexpected inhibition of the mitoBK_Ca_ channel by iberiotoxin (IbTx) was previously observed [62,63]. In case of mitoBK_Ca_ from glial cells, the dual orientation of the channels in the patch-clamp pipette was observed [64]. It is also important that the previous study showed that Tertiapin application from the matrix site did not inhibit the pH-sensitive channel in brain mitochondria. Moreover, this channel was also insensitive to ATP/Mg^2+^ and 5-HD [65]. This suggests that the TPNQ effect observed here is not coincidental.

Because the patch-clamp data were unclear, we decided to examine the topology of the channel with a biochemical approach. Our results based on PK accessibility to the ROMK protein suggest that the both N- and C-terminus are located in the mitochondrial matrix (Figure 5). In previous study published by Foster et al., Tertiapin Q added to the mitochondrial suspension was able to inhibit thallium uptake, thus suggesting the location of the inhibitor binding site facing the mitochondrial intermembrane space (IMS) [28]. The presence of the targeting sequence at the N-terminus of the protein [28] suggests that ROMK might be imported to the inner mitochondrial membrane via TOM and then TIM23 (translocase of the inner membrane) [66,67], which might support the topology of the channel with the C- and N-terminus on the matrix side. Interestingly, in the study describing the small conductance calcium-activated potassium channels (SK2) channel in mitochondria of HT22 cells, the PK treatment of swollen mitochondria gave the same result—the channel protein was digested only after mitochondria solubilization with Triton X-100 [68]. However, we propose that the topology of the channel is the opposite (with the vestibule facing mitochondrial matrix), and this conclusion is based on an electrophysiological experiment with whole-mitoplast patch-clamp. It is clear that the important question of mitoK_ATP_ topology remains controversial, and additional experiments are required to resolve this issue.

Though our study was limited and based on the cardiomyoblast cell line application of a unique electrophysiological technique, it provides good evidence for ROMK as a possible component of the mitoK_ATP_. However, cardioprotective mechanisms activated by K_ATP_ channel openers are complex phenomena. Previous data have suggested an interplay between the plasma membrane and the sarcoplasmic and mitochondrial K_ATP_ channels [69]. Moreover, the effects of K_ATP_ channel openers seem to be tissue dependent due to the differential expression of the components of the K_ATP_ channels [70]. Therefore, additional studies based on more complex models, including primary cardiomycytes are required in order to achieve further benefits from our discovery. 

## 4. Materials and Methods

### 4.1. H9c2 WT and H9c2 OE ROMK2 Cells and Isolation of Mitochondria

The heart-derived H9c2 WT cell line was purchased from ATCC. The H9c2 cell line which stably overexpressed the human ROMK2 protein with the V5 tag on the C-terminus (H9c2 OE ROMK2) was the cell line previously described by Foster and colleagues [28]. The H9c2 OE ROMK2 cell line was developed using gene encoding for human KCNJ1 (I.M.A.G.E. consortium, clone ID 30915211) [28]. Cells were cultured in standard 75 cm^2^ cell culture flasks (Thermo Fisher Scientific, Waltham, MA, USA) in a DMEM medium (Lonza) supplemented with 10% FBS, 2 mM of L-glutamine, 100 U/mL of penicillin, and 100 mg/mL of streptomycin at 37 °C in a cell incubator with a humidified atmosphere with 5% CO_2_. The medium for the ROMK OE cells was additionally supplemented with blasticidin. The cells were fed and re-seeded twice a week. For our study, we used cells from passages 10 to 30. Mitochondria were prepared as previously described [63]. In brief, cells were collected in a PBS medium and centrifuged at 800× *g* for 10 min. The cell pellet was resuspended and homogenized in a preparation solution (250 mM of sucrose, 5 mM of HEPES, pH = 7.2). To isolate the mitochondria, the homogenate was centrifuged at 9200× *g* (10 min). The pellet was then suspended and centrifuged at 790× *g* (10 min). The supernatant was transferred to a new tube and centrifuged at 9200× *g* for 10 min. The pelleted mitochondria were then resuspended in an isotonic solution (150 mM of KCl, 10 mM of HEPES, pH = 7.2). All procedures were performed at 4 °C in a cooling centrifuge, and the tubes and homogenizer were cooled on ice.

### 4.2. Patch-Clamp Experiments

Patch-clamp experiments using mitoplasts were performed as described previously [34,63]. Figure 1 shows a scheme of the patch-clamp experiments. Mitochondria isolated from cells were placed in a hypotonic solution (5 mM of HEPES, 200 μM of CaCl_2_, pH = 7.2) for approximately 1 min to induce swelling and breakage of the outer membrane. Then, a hypertonic solution (750 mM of KCl, 30 mM of HEPES, 200 µM of CaCl_2_, pH = 7.2) was added to restore the isotonicity of the medium. The patch-clamp pipette was filled with an isotonic solution containing 150 mM of KCl, 10 mM of HEPES, and 200 µM of CaCl_2_ at pH = 7.2. The electrical connection was made using Ag/AgCl electrodes and an agar salt bridge (3 M KCl) as the ground electrode.

The current was recorded using a patch-clamp amplifier (Axopatch 200B, Molecular Devices Corporation, Sunnyvale, CA, USA). The pipettes, made of borosilicate glass, had a resistance of 10–20 MΩ and were pulled using a Flaming/Brown puller. The currents were low-pass filtered at 1 kHz and sampled at a frequency of 100 kHz. The traces of the experiments were recorded in the single-channel mode. The ionic current was measured, if not otherwise mentioned, in a symmetric 150/150 mM KCl isotonic solution with a 200 µM CaCl_2_ concentration.

Mitoplasts are easily recognizable due to their size, round shape, and transparency, which distinguish these structures from cellular debris also present in the preparation. To apply the modulators of the channels, we used a perfusion system containing a holder with a glass tube connected to a peristaltic pump via tubing. The mitoplasts at the tip of the measuring pipette were transferred into the openings of a larger pipe system, and their outer faces were rinsed with the solutions of the channel modulators. The experiments were carried out in patch-clamp inside–out mode.

The illustrated channel recordings are representative of the most frequently observed conductance for the given condition. The conductance of the channel was calculated from the current–voltage relationship. The channel open probability (P_o_, open probability) was determined using the single-channel search mode of the Clampfit 10.2 software (Molecular Devices, San Jose, CA, USA). The results are presented as the means ± SD obtained from at least three independent experiments. An unpaired two-tailed Student’s t-test was used to identify significant differences; in particular, differences were considered to be statistically significant if * *p* < 0.05, ** *p* < 0.01, or *** *p* < 0.001.

### 4.3. Transient Transfection and Fluorescence Staining of H9c2 Cells

The H9c2 WT cells were seeded on a 4-chamber glass bottom dish (Cellvis). After overnight incubation, the cells were transiently transfected with plasmid encoding ROMK2 fused with GFP. Transfection was performed using a Fugene reagent (Promega, Madison, WI, USA). Forty-eight hours after transfection, the mitochondria were additionally stained using 100 nM Mitored (Sigma, St. Louis, MO, USA). Confocal images of living cells were acquired using an Olympus FV 1200.

### 4.4. Proteinase K and Western Blot Analysis

Mitochondria were isolated as described above. Samples were separated into two equal parts and stored on ice. One part was incubated for 15 min in an ice-cold hypotonic buffer to induce swelling and outer membrane breakage, while the second sample was stored in an ice-cold isotonic solution. Additionally, a separate set of mitochondria was solubilized with 0.1% Triton-X 100. Next, half of all samples were treated with Proteinase K (15 µg) for 10 min. Digestion was stopped with 2 mM of phenylmethylsulfonyl fluoride (PMSF) (an inhibitor of the Proteinase K). Next, all samples (around 50 µg of protein each) were separated by 10% sodium Tris-Tricine gel electrophoresis (SDS-PAGE) and transferred onto Polyvinylidene Difluoride (PVDF) membranes (BioRad, Hercules, CA, USA). The membranes were exposed to an anti-ROMK antibody (Sigma-Aldrich, diluted 1:200). To ensure the integrity of mitoplast and the Proteinase K activity, the sample was exposed to anti-Tim23 (BD Biosciences, Franklin Lakes, NJ, USA, diluted 1:2000), anti-Tom20 (Sigma, diluted 1:500) and anti-mHSP75 (Abcam, 1:1000) antibodies. The blots were developed using a secondary anti-rabbit (GE Healthcare UK, Buckinghamshire, UK) or anti-mouse antibody (GE Healthcare UK,) coupled to horseradish peroxidase in conjunction with an enhanced chemiluminescence solution (GE Healthcare UK,). Experiments were performed from independent mitochondrial isolations (*n* = 5). Each mitochondrial isolation was performed from 25–30 mln cells.

## 5. Conclusions

In summary, in the present work, we provide the first direct single-channel patch clamp evidence that the ROMK2 channel is the pore-forming unit of mitoK_ATP_. However, our data do not exclude the possibility that other proteins apart from ROMK might be responsible for ion channel activity with mitoK_ATP_ properties. Our data also suggest the C- and N-terminus matrix localization of the ROMK2 channel in the inner mitochondrial membrane.

## Supplementary Materials

Supplementary materials can be found at https://www.mdpi.com/1422-0067/20/21/5323/s1.

## Abbreviations

5-HD	5-hydroxydecanoic acid
IMS	Mitochondrial intermembrane space
IPC	Ischemic preconditioning
MIM	Mitochondrial inner membrane
MOM	Mitochondrial outer membrane.
mitoK_ATP_	Mitochondrial ATP-sensitive potassium channel
mitoBK_Ca_	Mitochondrial large-conductance, calcium-regulated potassium channel
PK	Proteinase K
PTP	Permeability transition pore
ROMK	Renal outer medullary potassium channel
SUR	Sulfonylurea receptor
