# Supplementary material for: Single-Channel Properties of the ROMK-Pore-Forming Subunit of the Mitochondrial ATP-Sensitive Potassium Channel
Source: Int J Mol Sci. 2019 Oct 25;20(21):5323. doi: 10.3390/ijms20215323 (PMC6862428; doi:10.3390/ijms20215323)
Supplement: Supplementary file 1 [file ijms-20-05323-s001.pdf]

Single-channel properties of the ROMK-pore-forming subunit of the mitochondrial ATP-sensitive potassium channel

Michał Laskowski, Bartłomiej Augustynek, Piotr Bednarczyk, Monika Żochowska, Justyna Kalisz, Brian O'Rourke, Adam Szewczyk and Bogusz Kulawiak

Single mitoplast PCR

One of the critical steps in patch-clamp experiments is the selection of vesicles that are genuine mitoplasts. To prove that it is possible to visually distinguish between these vesicles and cellular debris, we performed a single-mitoplast PCR assay (Figure S1).

The single-mitoplast PCR technique takes advantage of the fact that mitochondria are the only organelles in animals, aside from nuclei, that contain their own DNA particles (mtDNA). These mtDNA nucleotide sequences are unique and differ in sequence from nuclear DNA.

In this assay, a single mitoplast that is normally used in a patch-clamp experiment serves as a template in a PCR reaction. Mitochondria used for this assay were isolated as described above, in the presence of DNase (Qiagen, Germany) added during the homogenization step to destroy any DNA that is not protected from the enzyme with a lipid bilayer(s). Once isolated, a vesicle recognized as a genuine mitoplast was picked out of the mitoplast suspension with a micropipette. The micropipette had a wider tip opening than pipettes used in patch-clamp experiments, so that intact mitoplasts could be sucked into the pipette. The mitoplast was transferred to a PCR tube with reaction mix (JumpStart RedTaq ready mix; primers; DMSO; H_2O) (Table S1). The sealed PCR tube was incubated at 95 °C for 10 min to inactivate any traces of DNase. The PCR tube was placed in an ultrasound water bath to facilitate disruption of the mitoplast, and the tube was moved to the thermocycler to perform the PCR reaction. The single-mitoplast PCR program did not differ from a standard PCR method.

To detect mtDNA, the primers (mtDNA) that amplify genes of one of the mitochondrially encoded tRNAs molecules were used. Potential contamination of nuclear DNA was evaluated with primers (nuc) that amplify the *GAPDH* gene (Tabble S2). DNA products resulting from single-mitoplast PCR reactions were separated using standard agarose gel electrophoresis.

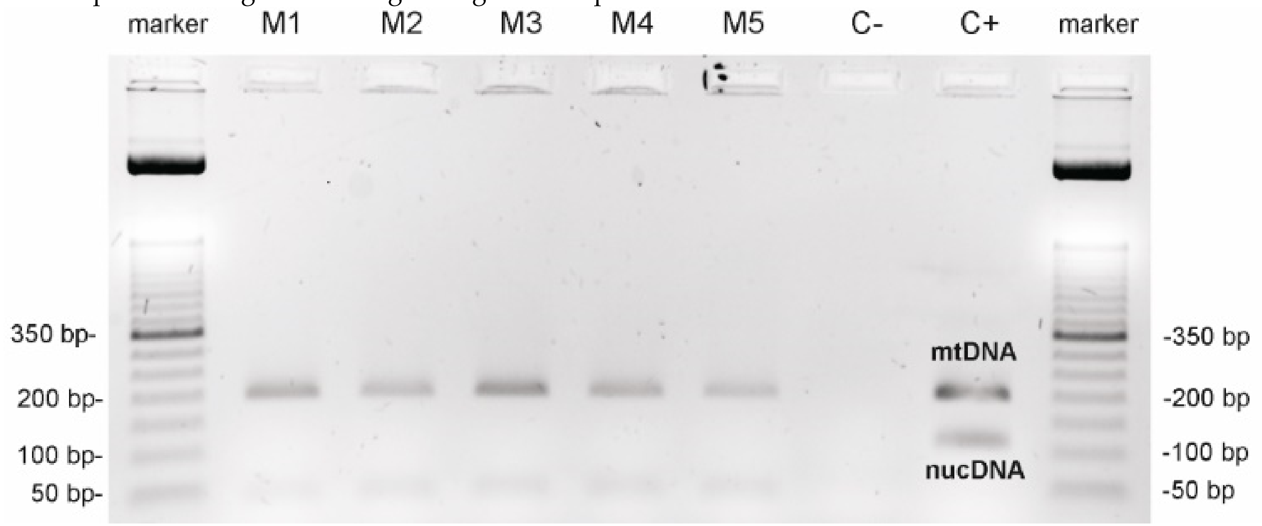

Figure S1. Single-mitoplast PCR. (M1-M5) reactions with mitoplasts added, (C^-) negative control: reaction with isotonic buffer added instead of mitoplast, (C^+) positive controls for mitochodrial DNA (mtDNA) and nuclear DNA (nucDNA). The experiment was performed on independent mitoplast isolations (*n* = 3).

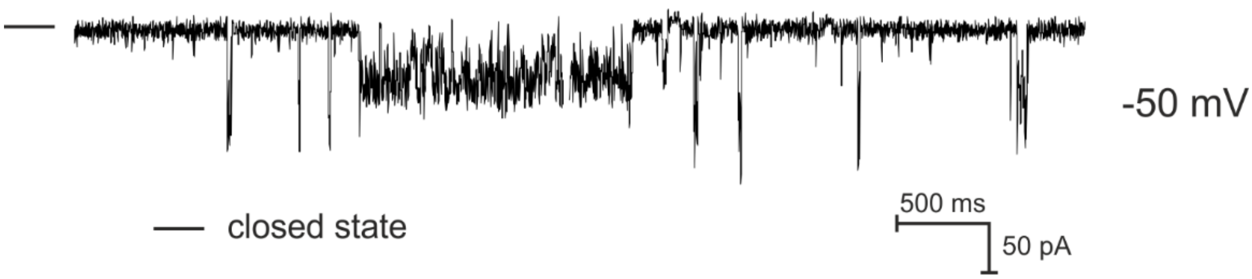

Figure S2. Typical single channel recording of high conductance channel (PTP-type). The channel was recorded using mitoplast isolated from H9c2 OE ROMK2.

Table S1. Composition of the PCR mix and thermal program of the reaction for single-mitoplast PCR.

Reagent	Volume	Thermal program	
Jump Start Red Taq Master Mix	12,5 µL	95 °C—3 min	
mtDNA primer forward	1 µL	95 °C—30 s	
mtDNA primer reverse	1 µL	56 °C—30 s	×35
nuc primer forward	1 µL	72 °C — 30 s	
nuc primer reverse	1 µL	72 °C — 5 min	
DMSO	0,5 µL	8 °C — forever	
H_2O	3 µL		
Mitoplast in isotonic buffer	~5 µL		
Total	25 µL		

Table S2. Sequence of the primers used in single-mitoplast PCR reactions along with the size of expected products and temperature of primers annealing.

Gene	Primers (5′–3′)	Size of the product [bp]	Annealing temperature [°C]
*mtDNA**	mtDNAfor:5′CACCCAAGAACAGGGTTTGT3′	107	56.0
	mtDNArev:5′TGGCCATGGGTATGTTGTTA3′		
nuc-GAPDH	GAPDHfor:5′CAAGGTCATCCATGACAACTTTG3′	495	56.0
	GAPDHrev:5’GTCCACCACCCTGTTGCTGTAG3’		

© 2019 by the authors. Submitted for possible open access publication under the terms and conditions of the Creative Commons Attribution (CC BY) license (http://creativecommons.org/licenses/by/4.0/).
